# Tumor Necrosis Factor-Alpha Induces Proangiogenic Profiling of Cardiosphere-Derived Cell Secretome and Increases Its Ability to Stimulate Angiogenic Properties of Endothelial Cells

**DOI:** 10.3390/ijms242316575

**Published:** 2023-11-21

**Authors:** Konstantin Dergilev, Ekaterina Zubkova, Alika Guseva, Zoya Tsokolaeva, Yulia Goltseva, Irina Beloglazova, Elizaveta Ratner, Alexander Andreev, Stanislav Partigulov, Mikhail Lepilin, Mikhail Menshikov, Yelena Parfyonova

**Affiliations:** 1 Federal State Budgetary, Institution National Medical Research Center of Cardiology Named after Academician E.I. Chazov, Ministry of Health of the Russian Federation, 121552 Moscow, Russia; 2 Federal Research and Clinical Center of Intensive Care Medicine and Rehabilitology, 141534 Moscow, Russia

**Keywords:** cardiosphere-derived cells, TNFalpha, cell secretome, angiogenesis

## Abstract

Ischemic heart disease and its complications, such as myocardial infarction and heart failure, are the leading causes of death in modern society. The adult heart innately lacks the capacity to regenerate the damaged myocardium after ischemic injury. Multiple lines of evidence indicated that stem-cell-based transplantation is one of the most promising treatments for damaged myocardial tissue. Different kinds of stem cells have their advantages for treating ischemic heart disease. One facet of their mechanism is the paracrine effect of the transplanted cells. Particularly promising are stem cells derived from cardiac tissue per se, referred to as cardiosphere-derived cells (CDCs), whose therapeutic effect is mediated by the paracrine mechanism through secretion of multiple bioactive molecules providing immunomodulatory, angiogenic, anti-fibrotic, and anti-inflammatory effects. Although secretome-based therapies are increasingly being used to treat various cardiac pathologies, many obstacles remain because of population heterogeneity, insufficient understanding of potential modulating compounds, and the principles of secretome regulation, which greatly limit the feasibility of this technology. In addition, components of the inflammatory microenvironment in ischemic myocardium may influence the secretome content of transplanted CDCs, thus altering the efficacy of cell therapy. In this work, we studied how Tumor necrosis factor alpha (TNFa), as a key component of the pro-inflammatory microenvironment in damaged myocardium from ischemic injury and heart failure, may affect the secretome content of CDCs and their angiogenic properties. We have shown for the first time that TNFa may act as a promising compound modulating the CDC secretome, which induces its profiling to enhance proangiogenic effects on endothelial cells. These results allow us to elucidate the underlying mechanisms of the impact of the inflammatory microenvironment on transplanted CDCs and may contribute to the optimization of CDC efficiency and the development of the technology for producing the CDC secretome with enhanced proangiogenic properties for cell-free therapy.

## 1. Introduction

Heart failure is the leading cause of morbidity and mortality, and it currently affects more than 60 million people worldwide [1]. One of the main causes is acute myocardial infarction, leading to massive cardiomyocyte death, fibrosis, pathological remodeling, and cardiac dysfunction [2]. The widely used treatment options are not able to replace the lost myocardial cells and activate repair processes that are necessary to prevent the development of heart failure. Meanwhile, it has now been strongly evidenced that stem/progenitor cells can mediate tissue repair by modulating the local environment, influencing the immune/inflammatory response, and supporting angiogenesis and cardioprotective activities [3]. The mechanism of such reparative effects appears to be largely due to the secretion of specific bioactive compounds rather than the direct differentiation of transplanted stem cells into host tissues, considering their low integration and survival rates after transplantation [4]. In accordance with this hypothesis, the paracrine effect is understood as a set of growth factors, cytokines, chemokines, and microvesicles orchestrating many biological activities and being secreted by progenitor cells.

Cardiosphere-derived cells (CDCs) are a promising cell type used for cell therapy [5,6]. CDCs are a combination of several progenitor cell populations derived from cultured myocardial explants that are cultured as 3D spheroids, resulting in the enhancement of their reparative properties [7,8,9]. Derived from spheroids, cardiosphere cells are capable of differentiating in cardiovascular lineages, but the key mechanism of their action is paracrine secretion of factors that can stimulate angiogenesis and cardiomyocyte proliferation/survival in recipient tissue [10,11,12]. CDCs exhibited a balanced profile of paracrine factor production compared with other commonly used progenitor cell types and showed the greatest functional effect in experimentally induced myocardial infarction [13]. Despite low engraftment and poor ability to differentiate in vivo, there is a consensus that their transplantation is safe and can improve cardiac functional performance, mainly through paracrine mechanisms [14,15]. Meanwhile, it is well known that transplanted cells respond to stress stimuli by adapting their secretion profile, increasing the production of cytokines and chemokines, which in turn may support cell survival in the injury zone either directly or indirectly through modulation of the immune response. Meanwhile, factors modulating cardiac cell secretome remain poorly understood, and mechanisms have not yet been clearly identified. This work aims to investigate how Tumor necrosis factor alpha (TNFa), a key factor in the pro-inflammatory microenvironment of infarcted myocardium, modulates the cardiosphere cell secretome.

## 2. Results

### 2.1. TNF Treatment Did Not Alter the Immunophenotype but May Enhance the Proliferative Properties of Cardiosphere-Derived Cells

**Figure 1:**
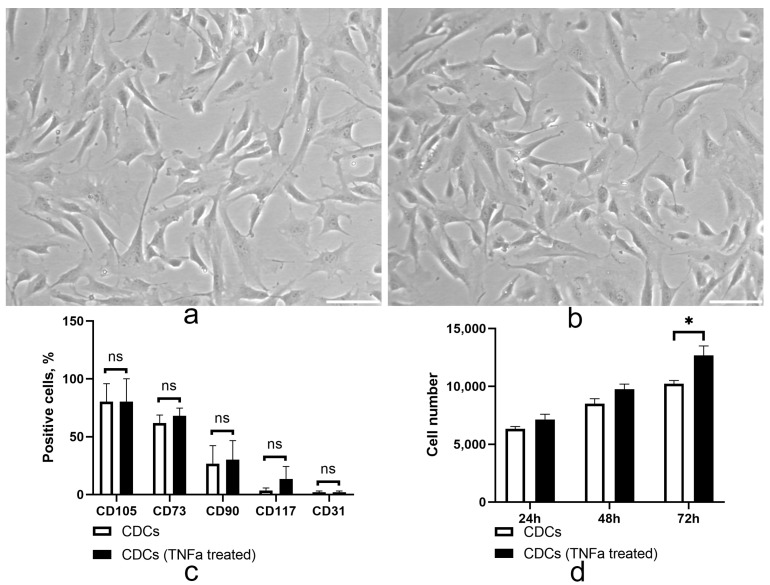
Phenotypic and proliferation analysis of untreated and TNFa-treated CDCs. The cells were cultured with or without TNFa (100 ng/mL). Representative images of the morphology of untreated (**a**) and TNFa-treated (**b**) CDCs. Phase-contrast light microscopy, scale bar represents 50 μm. (**c**) Flow cytometry analysis for specific immunophenotype markers: white histograms: CDCs; black histograms: TNFa-treated cells (100 ng/mL). Data are the average of triplicate experiments. (**d**) Proliferation analysis of untreated (white bars) and TNFa-treated (black bars) CDCs (100 ng/mL). Data are the average of triplicate experiments. Data are presented as mean ± SD; *—vs. untreated CDCs, *p* < 0.05; ns: not significant.

It is well known that MSC-like cells derived from different sources are sensitive to inflammatory factors [16], which can have a negative impact on cell properties after transplantation. We analyzed the effect of TNFa on the immunophenotype and proliferation properties of CDCs (Figure 1). We showed that treatment with TNFa did not alter the phenotype profile of CDCs (Figure 1c, Figure S1), maintaining expression of the key mesenchymal (CD105, CD73, CD90) and vascular progenitor cell markers (CD117, CD31). CDCs exhibited a mesenchymal spindle shape (Figure 1a,b), and TNFa treatment did not alter this characteristic cellular morphology. It was found that low concentrations of TNFa did not affect the ability of cardiosphere cells to divide. However, higher TNFa concentrations (100 ng/mL) enhanced the proliferative activity of CDCs cultured in vitro (Figure 1d).

### 2.2. TNFa Upregulates Angiogenic Proteins in Cardiosphere-Derived Cells

**Figure 2:**
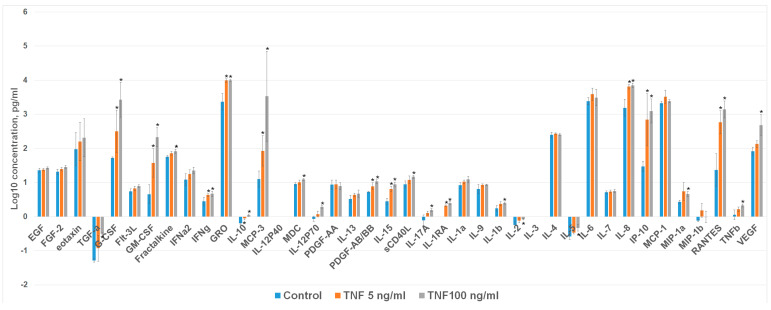
The cytokine/chemokine profile in the condition medium of CDCs. Concentrations of cytokines/chemokines were measured in conditioned medium obtained from untreated CDCs (blue bars) and CDCs treated with TNFa (5 and 100 ng/mL) (orange and gray bars, respectively). Data are the average of triplicate experiments. Data are presented as mean ± SD; *—vs. untreated CDCs (blue bar), *p* < 0.05.

To determine the effects of TNFa on the CDC secretome (Figure 2), protein profiles in the culture medium of cells were analyzed using the Multiplex Immunoassay (Magpix). Treatment of CDCs with TNFa enhanced the secretion of pro-angiogenic/-inflammatory factors G-CSF, GM-CSF, GRO1, IL8, IP10, MIP1a, MCP-3, and RANTES while not changing secretion of EGF, bFGF, IFNg, MDC, PDGFaa, IL1a, IL9, IL4, IL6, MCP1, and MIP1b. Incubation of CDCs with higher TNF concentration (100 ng/mL) significantly increased VEGF secretion compared to the case when the stimulant was used at a lower concentration.

### 2.3. The TNFa-Induced Secretome In Vitro Stimulates Proliferation and Proangiogenic Properties of Endothelial Cells

**Figure 3:**
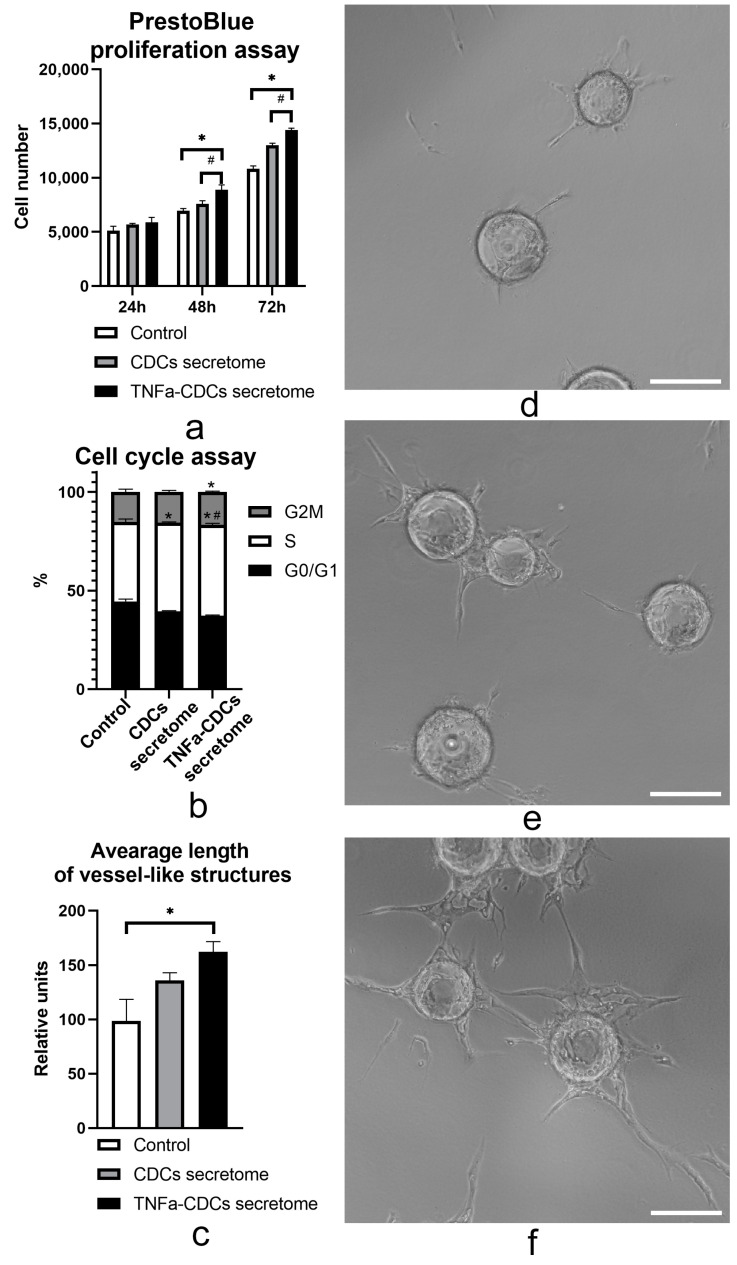
Effect of the conditioned media of CDCs on proliferation and angiogenesis of endothelial cells. (**a**) The secretome of TNFa-treated (100 ng/mL; black bar) CDCs increased endothelial cell proliferation. Data are the average of triplicate experiments and are presented as mean ± SD. *—vs. control medium (EBM + 2% FBS + 20% DMEM/F12; black bar), *p* < 0.05; #—vs. CDC secretome (EBM + 2% FBS + 20% secretome; gray bar), *p* < 0.05; (**b**) HUVECs were incubated for 48 h in control or conditioned mediums of CDCs, TNFa-CDCs (CDCs treated with TNFa 100 ng/mL). Cell cycle analysis was performed using flow cytometry; (**c**) Quantitative analysis of the average length of vessel-like structures formed by fibrin gel. *—vs. control medium (EGM2/ DMEM/F12 (1:1); white bar); Representative images of vessel-like structures formed by endothelial cells in control medium (**d**) and after treatment with conditioned media of CDCs (**e**), CDCs treated with TNFa (100 ng/mL) (**f**). Scale bar represents 200 μm.

Increased production of proangiogenic factors following TNFa cell treatment may play an important role in inducing angiogenesis through multiple mechanisms, including enhanced proliferation and angiogenic behavior of endothelial cells. To test this hypothesis, we investigated the effects of CDC secretome on the aforementioned properties of endothelial cells. PrestoBlue assay was conducted to determine whether the TNFa-induced secretome exhibited a promitogenic effect on human umbilical vein endothelial cells (HUVEC). We found (Figure 3a) that conditioned media obtained from untreated and TNFa-treated CDCs enhanced endothelial cell proliferation. In this case, the secretome of TNF-stimulated CDCs exerted a stronger promitogenic effect. Furthermore, we measured cellular DNA content by flow cytometry to determine cell cycle distribution. Compared to the control, the TNF-stimulated CDC secretome treatment exhibited a significantly increased proportion of HUVECs reaching the S and G2M stages (Figure 3b). We also found that the conditioned medium of CDCs treated with high doses of TNFa (100 ng/mL) promoted the stimulation of angiogenic behavior of endothelial cells in fibrin bead assay (Figure 3). We observed an increase in the average length of vessel-like structures formed in fibrin gel after stimulation of endothelial cells by conditioned medium of cells treated with TNFa (100 ng/mL) (Figure 3c–f). The effects of the secretome after stimulation of CDCs with a lower concentration of TNFa (5 ng/mL) were not statistically significant.

### 2.4. TNFa-Pretreated Cardiosphere-Derived Cells Stimulate Vascularization of Matrigel after Transplantation

**Figure 4:**
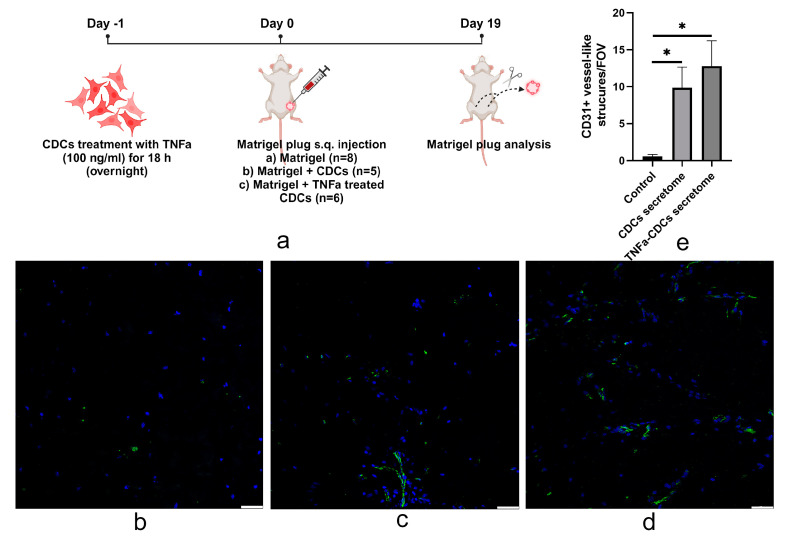
In vivo angiogenesis within Matrigel plugs. (**a**) Experimental design schematic (created with BioRender.com (https://app.biorender.com accessed on 16 November 2023)). Matrigels with untreated and TNFa-treated CDCs were injected subcutaneously. After 19 days, plugs were recovered by dissection. (**b**–**d**) the representative images of control (**b**) and Matrigel plugs with cells (with CDCs (**c**) and TNFa-treated CDCs (**d**)) stained with antibodies against endothelial marker CD31 (green). The nuclei were stained with DAPI (4′,6-diamidino-2-phenylindole). (**e**) Quantitative analysis of the number of capillary-like structures. *—vs. control (Matrigel; black bar), *p* < 0.05. Scale bar represents 50 μm.

We used the Matrigel plug angiogenesis assay to study the in vivo proangiogenic potential of TNFa secretome-induced cardiosphere cells. For this purpose, control cells (untreated) and TNFa-treated CDCs were mounted in Matrigel and transplanted subcutaneously into immunodeficient mice (Figure 4a). After 19 days post-transplantation, we found that the number of vessels formed after transplantation of each of the two cell types was more than 9-fold higher than when Matrigel was injected without cells (Figure 4b–e). When using TNFa-stimulated cells, a trend toward enhanced proangiogenic activity was observed compared with unstimulated CDCs; however, it was not statistically significant.

## 3. Discussion

Cardiosphere-derived cells are a promising tool for targeting the cardiac microenvironment and activating regenerative processes in the heart due to their pronounced immunosuppressive activity, as well as anti-apoptotic, anti-fibrotic, and proangiogenic properties mediated by paracrine mechanisms. Moreover, the therapeutic efficacy of exposure to the CDC secretome, implemented through the action of soluble factors as well as extracellular vesicles (EVs), transferring the endogenous molecules to salvage the injured neighboring cells by regulating apoptosis, inflammation, fibrosis, and angiogenesis can vary significantly in response to inflammatory factors. Immune cells expressing various cytokines, chemokines, growth factors, and adhesion molecules are present in the heart, thus either directly or indirectly regulating cell viability, differentiation capacity, and secretion in their microenvironment.

In this study, we investigated the effects of a strong pro-inflammatory factor, TNFa, on CDCs. The results obtained have shown for the first time that: (1) TNFa did not induce changes in the phenotype but may enhance proliferative properties of CDCs; (2) TNFa enhanced proangiogenic /-inflammatory characteristics of the CDC secretome; (3) the secretome of TNFa-treated CDCs increased the proliferative capacity of endothelial cells; and (4) untreated and TNFa-treated CDCs induced significant Matrigel plug vascularization in mice. Only an insignificant trend toward more pronounced vascularization was noticed in Matrigels with TNFa-treated CDCs.

It is well known that exposure to ischemia, viral and bacterial infection causes the rapid release of large amounts of TNFa from macrophages, lymphoids, mast cells, fibroblasts, and endothelial cells [17,18,19,20]. The development of coronary obstruction is also associated with an increase in TNFa concentrations mediated by nitric oxide (NO) produced by endothelial NO synthase (eNOS) [21,22,23,24]. In addition, the development of inflammation and increased TNFa levels are associated with the pathogenesis of heart failure and act as an indicator of myocardial dysfunction, adverse remodeling, and increased risk of mortality [25,26,27]. Consequently, TNFa acts as an important component of the cardiac cellular microenvironment, providing a fine balance between normal and pathologic states [28]. Meanwhile, pro-inflammatory signaling and chronic inflammation cause growth arrest and DNA damage, which is critical for cell integration, survival, and functional activity after transplantation [29,30]. This sophisticated pathway of regulation is implemented through the interaction between TNFa and specialized receptors. Earlier studies show that CDCs express TNFR1 and TNFR2 receptors on their surface [31] and represent single transmembrane glycoproteins with extracellular TNFa binding domains characterized by four tandem-repeated cysteine-rich motifs [32], suggesting that TNFa may affect CDC functions. In this study, we have found that treatment of CDCs by TNFa did not affect the morphology and immunophenotype of the cells but stimulated cell proliferation when applied at high doses (100 ng/mL). Our previous results on adipose tissue mesenchymal stem cells (MSCs) suggested that this effect may be mediated by ROS production and activation of PI3K pathways [33]. In the present study, we have shown that TNFa significantly upregulates the secretion of numerous proangiogenic and inflammatory factors by CDCs, including vascular endothelial growth factor (VEGF), the key mediator of angiogenesis stimulating proliferation, migration, and angiogenic behavior of endothelial cells [34,35], as well as inflammatory cytokines with angiogenic properties such as Rantes [36] and G-CSF [37], GM-CSF [38], GRO1 [39], IL8 [40], and IP10 [41].

Acting together, all these factors may provide proangiogenic profiling of TNFa-treated CDC secretome, corresponding to a promising strategy for improving the regenerative effects of cells by modulating the components of their secretome. This approach is based on the optimization of cell culture conditions in the presence of an activating stimulus (“priming”) in order to optimize the properties of their secretome. For this purpose, it is proposed to use different compounds such as lipopolysaccharide (LPS), polyinosinic: polycytidylic acid (Poly (I: C), curcumin, oxytocin, melatonin, cytokines (IL17A, IL1β, FGF2, TNFa, and IFNγ, as well as culturing under hypoxia conditions [42,43]. This approach is well-known for MSCs from various sources but remains poorly investigated in CDCs. Our findings suggest that exposure of CDCs to TNFa induced proangiogenic changes in their secretome. As a result of TNFa priming, the CDC secretome enhanced the proliferation and angiogenic behavior of endothelial cells in vitro. However, in vivo, both untreated and TNFa-polarized CDCs substantially enhanced subcutaneous Matrigel vascularization with no significant difference between them. At the same time, in our previous study, pretreatment of adipose tissue MSCs with TNFa accelerated blood flow recovery, which was accompanied by increased arteriole density and reduction in necrosis in the mouse hind limb ischemia model [33]. Subcutaneous transplantation of untreated CDCs probably induced maximal vascularization of the Matrigel plug; therefore, the effect of TNFa-treated CDCs cannot be detected in this in vivo angiogenesis model. Another model, such as the mouse hind limb ischemia model, may provide evidence for the increased angiogenic potential of TNFa-treated CDCs.

A significant limitation of our study is the relatively short-term impact of TNFa on cardiosphere cells and the subsequent effect of their secretome on the angiogenic properties of endothelial cells. Thus, we cannot predict how prolonged exposure to TNFa or its combination with other pro-inflammatory factors might affect the survival and paracrine secretion capacity of cardiosphere cells. In addition, it remains unknown what changes in the microRNA profile (which are packed in extracellular vesicles and participate in the implementation of reparative effects of the secretome) occur after treatment with TNFa. Another limitation is that multiple signaling mechanisms may be synergistically triggered through the effects of individual secreted proteins that have their specific targets. Identification of proteins, their targets, and signaling mechanisms, as well as the feasibility of combined effects, is the subject of further research aimed at developing an optimized secretome product with optimal therapeutic properties.

In general, the results of these studies proved that preconditioning of CDCs with TNFa promoted proangiogenic profiling of their secretome, making it more angiogenic in in vitro models of angiogenesis. These findings provide a basis for further studies aiming to develop novel types of cell-free products based on the CDC secretome for ischemic heart disease treatment.

## 4. Materials and Methods

### 4.1. Cardiosphere, Cardiosphere-Derived Cell Isolation, and TNFa Treatments

Samples of the right atrial appendages were obtained from 18 male patients during aortocoronary bypass surgery performed in the Department of Cardiovascular Surgery of Federal State Budgetary Institution National Medical Research Center of Cardiology Named after Academician E.I. Chazov after informed consent had been obtained and the study conduct was approved by the Ethical Committee of the study site (permit #271, (27 September 2021) and study protocol addendum (permit #290, (29 May 2023)). Cell isolation was performed according to the optimized protocol described previously [14]. Briefly, the specimens were excised, minced, and digested with 0.2% type A collagenase to obtain cardiosphere-forming cells. Cells were then seeded onto low-adhesion culture dishes, coated with poly(2-HEMA) (12 mg/mL) (Sigma-Aldrich, Saint Louis, MO, USA), to generate cardiospheres with growth medium containing DMEM/F12, 10% FBS, 20 ng/mL epidermal growth factor (Peprotech, London, UK), and 50 ng/mL basic fibroblast growth factor (Peprotech, London, UK). Cardiospheres were selectively picked, plated onto fibronectin-coated culture dishes to obtain CDCs, and grown as an adherent culture for no more than five passages. Cells were treated with TNFa (5 and 100 ng/mL) for 18 h (overnight) and washed thrice; the culture medium was replaced with DMEM/F12 without supplements, and the conditioned medium was harvested after 24 h.

### 4.2. Flow Cytometry

To determine immunophenotype, untreated and TNFa-treated CDCs were washed twice with ice-cold PBS, resuspended in ice-cold PBS supplemented with 1% BSA and incubated with primary labeled (anti-CD105 (Becton Dickenson, Franklin Lakes, NJ, USA; #560839; 1:100), anti-CD73 (Becton Dickenson, Franklin Lakes, NJ, USA; #550257; 1:100), anti-CD90 (Becton Dickenson, Franklin Lakes, NJ, USA; #555596; 1:100)), anti-CD117 (Becton Dickenson, Franklin Lakes, NJ, USA; #567132; 1:100), and anti-CD31 (Becton Dickenson, Franklin Lakes, NJ, USA; #555446; 1:100)) or isotype control antibodies (Becton Dickenson, Franklin Lakes, NJ, USA; #554680; 1:100) for 30 min at 4 °C. The stained cells were washed with ice-cold PBS and fixed in PBS supplemented with 1% paraformaldehyde. Flow cytometry analysis was performed using a FACS Aria III system (Becton Dickinson, Franklin Lakes, NJ, USA).

### 4.3. PrestoBlue Assay

Cell viability was tested using the Presto Blue assay as described previously [15] with modifications. CDCs or endothelial cells (HUVECs were gifted by Dr. Olga Antonova (Cell Adhesion department, Institution National Medical Research Center of Cardiology Named after Academician E.I. Chazov) were plated in 96-well culture plates (5 × 10^3^ cells/well). Cells were incubated for 24, 48, and 72 h, and 10 µL of fluorescent dye PrestoBlue™ Cell Viability Reagent (Invitrogen) was then added to the culture medium for another 1 hr. Fluorescence intensity was measured on a Victor X3 spectrophotometer (Perkin Elmer, Waltham, MA, USA) at a wavelength of 570 nm, and the number of cells per well was calculated using a plotted standard curve.

### 4.4. Cell Cycle Assay

Cell Cycle Assay Kit (Elabscience, Wuhan, China) was used for cell cycle analysis according to the manufacturer’s specifications. Briefly, HUVECs were incubated for 48 h in control (EBM + 2% FBS + 20% DMEM/F12) or conditioned mediums (EBM + 2% FBS + 20% secretome) of CDCs, TNFa-treated (100 ng/mL) CDCs. HUVECs were dispersed with 0.025% Trypsin-EDTA, then washed with phosphate-buffered saline, suspended in PBS to generate the single suspension, and fixed in methanol overnight at −20 °C. The methanol was removed by centrifuge, and HUVECs were suspended in PBS containing RNase A for 30 min at 37 °C and then stained with propidium iodide. DNA fluorescence was measured on a FACS Aria III system (Becton Dickinson, USA). Histograms of DNA content were analyzed using Modfit LT to determine fractions of the population in each phase of the cell cycle.

### 4.5. Magpix Analysis

A MILLIPLEX MAP 41 Human Cytokine/Chemokine Magnetic Bead Panel human multiplex kit (Merck, Steinheim, Germany) for simultaneous quantitative determination of 41 analytes in a single sample and the MAGPIX^®^ System (Luminex -Merck, Siegen, Germany) were used according to manufacturer’s recommendations to determine chemokine/cytokine profiles of control and TNF-treated CDCs derived from different donors.

### 4.6. Fibrin Gel Bead Angiogenesis Assay

The effect of CDC pretreatment with TNFa on angiogenesis was analyzed using fibrin gel bead assay as previously described [33]. HUVECs at passage 3 were cultured on Cytodex microbeads (Sigma-Aldrich, Saint Louis, MO, USA). Beads with attached cells were labeled with 5 μM CellTracker™ Green CMFDA (Life Technologies, Carlsbad, CA, USA) and placed in a fibrin clot at a rate of 250 beads/well of a 24-well plate. The medium was then added to the wells as follows: (1) EGM2 culture medium; (2) EGM2 /DMEM/F12 without supplement (1:1); (3) EGM2 /CDCs secretome (1:1); (4) EGM2 /TNFa (5 ng/mL) treated CDCs secretome (1:1); and (5) EGM2 /TNFa (100 ng/mL) treated CDC secretome (1:1). After 5 days of incubation, gels were fixed and imaged on a Leica Stellaris 5 confocal microscope (Leica, Wetzlar, Germany). Average sprout lengths per bead were measured using the ImageJ software (https://imagej.nih.gov/ij/download.html accessed on 16 November 2023).

### 4.7. Matrigel Plug Assay

All the in vivo studies were performed in accordance with the Code of Practice for the Housing and Care of Animals Used in Scientific Procedures, American Association for Laboratory Animal Science and Institute of Experimental Cardiology guidelines. Animal procedures were approved by the Ethics Board of Institutional Animal Care and Use Committee of National Medical Research Center of Cardiology Named after Academician E.I. Chazov (permit #LA/28.07.2023 (28 July 2023)). In order to evaluate the angiogenic potential of control and TNFa-pretreated CDCs, Matrigel plug assay was performed in eight-week-old BALB/c nude mice (Bioresource Collection of SPF Laboratory Rodents for Fundamental, Biomedical and Pharmacological, Moscow region, Pushchino, Russia). For the Matrigel plug assay, mice were randomly divided into three groups: (1) growth factor-reduced Matrigel (BD Bioscience) was mixed with (DMEM/F12 without supplement alone); (2) growth factor-reduced Matrigel in combination with control CDCs (4 × 10^5^ cells resuspended in DMEM/F12); (3) growth factor-reduced Matrigel in combination with CDCs pretreated with TNFa (4 × 10^5^ cells resuspended in DMEM/F12) and subcutaneously injected into both flanks of mice. Nineteen days later, Matrigel plugs were harvested and processed for vessel quantification. To assess the vascularization of the Matrigel plugs, cryosections were stained with antibodies against endothelial cell marker CD31. Sections were fixed in ice-cold acetone for 20 min, air-dried, and washed in PBS. After washing, slides were blocked by 10% normal donkey serum for 30 min. Antibodies were diluted in blocking solution (1% BSA in PBS), and sections were incubated with rat anti-mouse CD31 antibody (BD Pharmingen, Franklin Lakes, NJ, USA; #553370, 1:100). The slides were then washed in PBS and incubated in a mixture of goat anti-rat AlexaFluor488-conjugated antibodies (Life Technologies, USA) for 40 min. At the end of incubation, nuclei were stained with DAPI, and sections were mounted under coverslips. Microphotographs were taken under 200× magnification. Vessel counts were performed manually using the Image J software.

### 4.8. Statistical Analysis

Values are presented as mean ± SD. The Statistica 8.0 statistical software was used for data analysis. Statistically significant differences between the two groups were determined using the Mann–Whitney U test, depending on the sample distribution profile. Multiple groups were compared using ANOVA with Bonferroni correction where required. *p*-values less than 0.05 were considered indicative of significance.

## Data Availability

Data is contained within the article and supplementary material.

## Supplementary Materials

The supporting information can be downloaded at: https://www.mdpi.com/article/10.3390/ijms242316575/s1.
