# Supplementary material for: Tumor Necrosis Factor-Alpha Induces Proangiogenic Profiling of Cardiosphere-Derived Cell Secretome and Increases Its Ability to Stimulate Angiogenic Properties of Endothelial Cells
Source: Int J Mol Sci. 2023 Nov 21;24(23):16575. doi: 10.3390/ijms242316575 (PMC10706276; doi:10.3390/ijms242316575)
Supplement: Supplementary file 1 [file ijms-24-16575-s001.zip › ijms-2665944-supplementary.pdf]

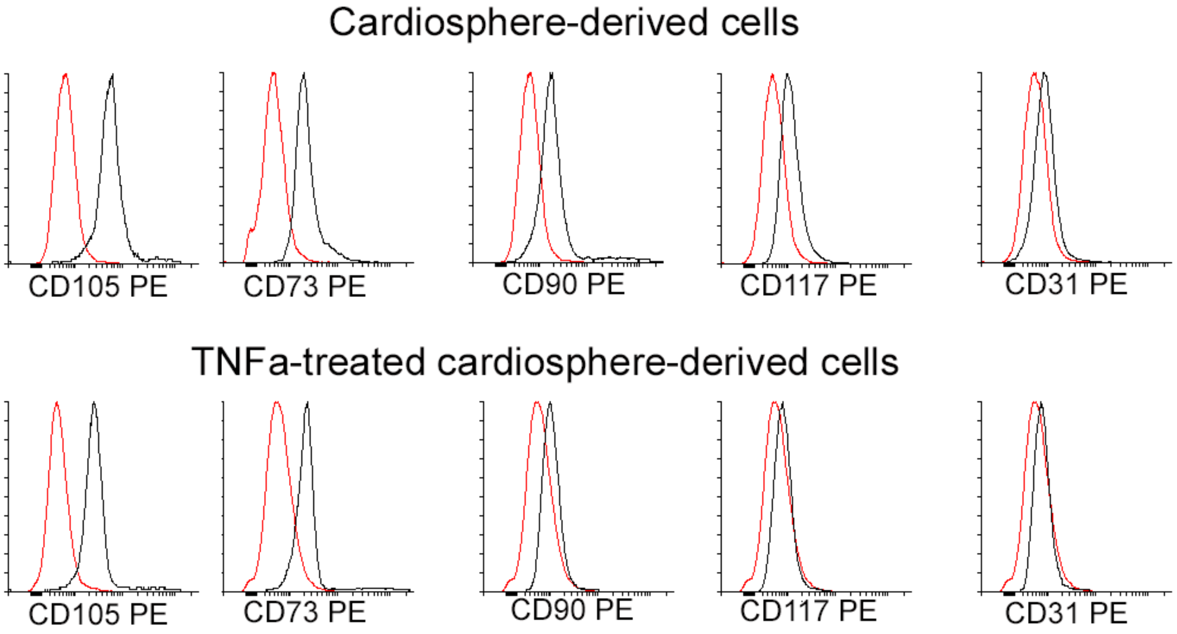

Figure S1. Representative histogram of the flow cytometry analysis of the MSC-related surface markers in CDCs and TNFa-treated CDCs.
